# Generalists and Specialists: A New View of How MHC Class I Molecules Fight Infectious Pathogens

**DOI:** 10.1016/j.it.2018.01.001

**Published:** 2018-05

**Authors:** Jim Kaufman

**Affiliations:** 1 University of Cambridge, Department of Pathology, Tennis Court Road, Cambridge CB2 1QP, UK; 2 University of Cambridge, Department of Veterinary Medicine, Madingley Road, Cambridge CB2 0ES, UK

## Abstract

In comparison with the major histocompatibility complexes (MHCs) of typical mammals, the chicken MHC is simple and compact with a single dominantly expressed class I molecule that can determine the immune response. In addition to providing useful information for the poultry industry and allowing insights into the evolution of the adaptive immune system, the simplicity of the chicken MHC has allowed the discovery of phenomena that are more difficult to discern in the more complicated mammalian systems. This review discusses the new concept that poorly expressed promiscuous class I alleles act as generalists to protect against a wide variety of infectious pathogens, while highly expressed fastidious class I alleles can act as specialists to protect against new and dangerous pathogens.

## Highlights

A broad overview of classical MHC I expression and bound peptides reveals an inverse correlation between repertoire breadth and cell-surface expression in some chicken and human alleles.

Several chicken class I alleles with wide peptide-binding repertoires (promiscuity) are associated with resistance to a variety of common diseases.

Conversely, a narrow peptide-binding repertoire (fastidiousness) in some human HLA-B alleles is associated with resistance to HIV progression.

Cell-surface expression of some classical class I alleles depends on the regulation of translocation to the cell surface rather than of transcription or translation. MHC translocation is influenced by peptide translocation in chickens and by tapasin interaction in humans.

## Insights from Studying a Simpler System

An enormous body of knowledge about the MHC and MHC molecules has been amassed over the past 50 years, mostly due to work on humans and important biomedical model species like mice [1]. This information is extremely detailed, complex but well integrated, and crucially important both for basic scientific understanding of immune and autoimmune responses and for practical medical applications, including transplantation 2, 3. What is the point of trying to understand the MHC in non-mammalian vertebrates, when there is such rich and relevant knowledge for placental mammals?

Besides the obvious importance to disease resistance and vaccination in poultry 4, 5, research into the chicken MHC has led to novel insights about the evolution of the adaptive immune system 6, 7, 8, 9. This short review highlights a third advantage: how the simplicity (at least in some senses) of the chicken MHC has permitted the discovery and/or study of phenomena that have been more difficult to discern in the complex MHC biology of humans and other typical mammals.

## Resistance to Infectious Disease

It is generally accepted that the high level of allelic polymorphism of MHC classical class I and class II genes is driven by a molecular arms race with pathogens 10, 11. An expectation from this relationship is that particular MHC alleles would confer resistance or susceptibility to particular infectious pathogens. The human MHC does have many strong genetic associations with autoimmune disease, but the reported associations with infectious disease are much weaker 2, 12. In essence, it has taken the best immunologists, epidemiologists, and geneticists decades to provide convincing evidence for such genetic associations. The best-studied example is the slow progression of HIV infection to AIDS conferred by the presence of certain HLA-B alleles as well as the cell-surface expression levels of HLA-C alleles 13, 14.

By contrast, decades ago poultry immunologists were already stumbling over extremely strong associations between the B blood group and resistance to a variety of economically important infectious diseases [15]. The MHC encoding classical class I and class II molecules is one region (the so-called BF-BL region) in this B locus [16]; nearby are regions with CD1 genes, TRIM genes, and the mysterious BG genes that have some similarities to butyrophilins 4, 5. Initially, these associations were with responses to oncogenic viral diseases such as Marek’s disease and Rous sarcoma, with the B locus determining life or death for individual chickens. Now there is a long list of viruses, bacteria, and even parasites that have significant associations with the BF-BL region 4, 5, 17, 18.

## A Minimal Essential MHC with a Single Dominantly Expressed Class I Molecule

**Figure 1:**
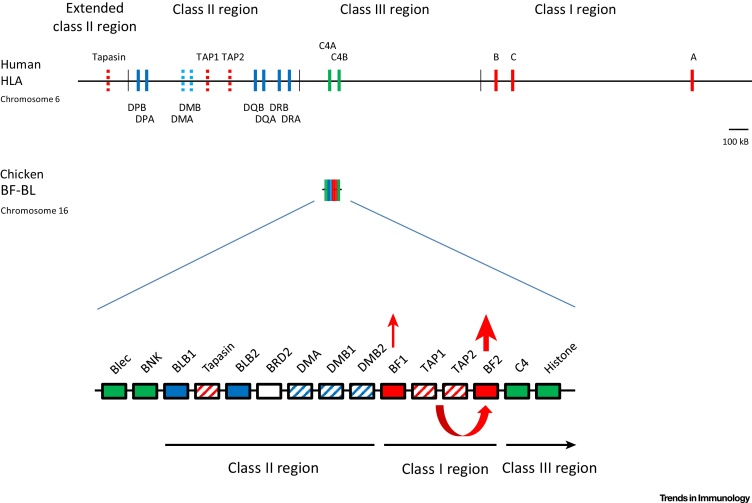
The Chicken Major Histocompatibility Complex (MHC) (BF-BL region) Is Much Smaller and Simpler than the Human MHC (HLA Locus), with a Single Dominantly Expressed Class I Molecule due to Coevolution with Peptide-Loading Genes. Colored vertical lines or boxes indicate genes, with names above or below; thin vertical lines indicate regions, with names above or below; location is roughly to scale, with the length of approximately 100 kB indicated for the upper portion of the figure. The thickness of arrows pointing up indicates the level of expression; coevolution between the TAP genes and the BF2 class I gene is indicated by a curved arrow beneath the genes. Red, genes from class I system; blue, genes from class II system; green, genes from class III or other regions. Solid colors indicate classical genes while striped colors indicate genes involved in peptide loading. Modified from 16, 91.

Compared with the MHC of typical mammals, the BF-BL region of chickens (also sometimes called the ‘classical MHC’ or the ‘core MHC’) is compact, simple, and arranged differently (Figure 1), with two class II B (so-called BLB) genes flanking the tapasin gene located near the DM genes followed by a pair of class I (so-called BF) genes that flank the TAP genes and, finally, the class III region genes [16]. Moreover, no recombination within the BF-BL region has been observed in experiments 19, 20, 21, 22, although comparison of haplotypes shows that there has been some recombination over unknown spans of time 23, 24, 25. Also, the genes involved in peptide loading (tapasin, TAP, and DM) are all highly polymorphic, with each BF-BL haplotype generally having a unique set of alleles 24, 26, 27, 28. The monomorphic DR-like class II A gene (BLA) is located some 5 cM away [29], the monomorphic β_2_-microglobulin (β_2_m) gene is on a different chromosome 30, 31, and inducible proteasome (LMP/PSMB) genes have not been found in the genome [32]. Thus, the polymorphic classical class I and class II B genes are in strong linkage disequilibrium with polymorphic peptide-loading genes, leading to relatively stable MHC haplotypes of polymorphic coevolving genes 33, 34.

This coevolution is clearly seen in the chicken class I system, in which the specificity of peptide translocation by the TAP alleles correlates with the peptide motif of the class I molecule encoded by the BF2 (but not the BF1) gene 27, 34, 35. Thus, the BF2 class I molecule receives lots of peptides whereas the BF1 molecule gets far fewer peptides and might be expected to have become much less important for antigen presentation. The BF1 gene has suffered deletions and insertions leading to far less expression at the RNA and protein levels than the BF2 gene 36, 37. Most importantly, the peptides presented by the dominantly expressed BF2 molecule can explain the immune response to certain economically important viruses and vaccines 37, 38, 39.

Such a system of coevolving alleles is not found in most placental mammals. In humans and other typical mammals, the antigen-processing and peptide-loading genes are located in the class II and extended class II regions, far from the class I genes that they serve [40]. Thus, alleles of antigen-processing and peptide-loading genes that were advantageous for any particular class I allele would relatively rapidly be switched by recombination [41] and any advantage lost. There are few sequence alleles of TAP, tapasin, and inducible proteasome components and these appear to be functionally monomorphic 42, 43, 44, working as average best fits to provide peptides for all class I molecules regardless of locus or allele. This situation allows a multigene family of class I genes all of which are (or can be, for HLA-C) relatively well expressed. To be clear, there are mammals (like rats) for which the classical class I genes have moved close to the antigen-processing and peptide-loading genes, with the result that one of the TAP genes is oligomorphic and coevolves with the class I molecules [45].

**Figure 2:**
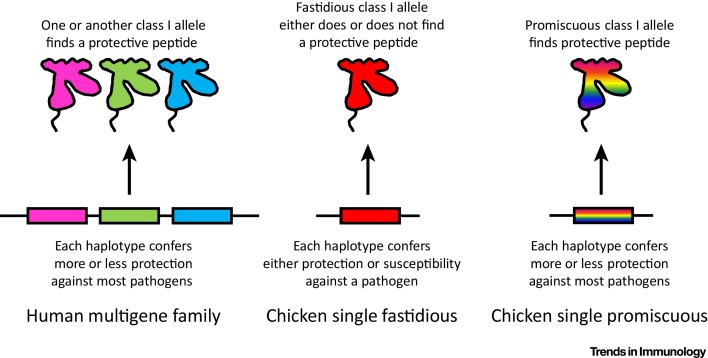
Compared with Mammals, the Major Histocompatibility Complex (MHC) of Chickens Has Strong Genetic Associations with Resistance and Susceptibility to Infectious Diseases. (A) A multigene family in the human MHC can encode multiple fastidious class I molecules each of which has a chance to find a protective peptide. Altogether the typical human MHC haplotype confers more or less resistance to most pathogens, a situation that reads out as a weak genetic association (since there is not much difference between haplotypes). (B) By contrast, the single dominantly expressed class I molecule encoded by the chicken MHC can have a fastidious peptide motif that may or may not find a protective peptide from any given pathogen, a situation that reads out as strong genetic associations (since there can be enormous differences between haplotypes). (C) However, the single dominantly expressed class I molecule encoded by the chicken MHC can have a promiscuous peptide motif capable of binding a wide variety of peptides (much like the multigene family of human class I molecules acting together). Comparison of two promiscuous alleles may read out as a weak genetic association (since there is not much difference between them) but comparison of a fastidious allele with a promiscuous allele in chickens may give strong genetic associations. Modified extensively from [33].

The difference in the number of class I loci that encode well-expressed class I molecules provides at least part of the explanation for the difference between the human and the chicken MHC in genetic association with infectious disease 4, 33. In humans, if one class I molecule does not bind a protective peptide, it is likely that another one will, so that overall most MHC haplotypes confer more or less resistance to most pathogens, which reads out as low genetic association. In chickens, the single dominantly expressed class I molecule either finds a protective peptide from a particular pathogen or does not, and this life-and-death difference between haplotypes reads out as strong genetic association (Figure 2). Thus, the simplicity of the chicken MHC has allowed greater appreciation of this phenomenon of resistance to infectious disease.

## Evolution of the MHC

Why do chickens and typical mammals differ in the genomic organization of their MHCs, if the end result can be so dire for an individual chicken? The salient features of the chicken MHC class I system can be found in many if not most non-mammalian vertebrates 6, 9, 46. For example, ducks have polymorphic TAP genes next to five class I genes only one of which is expressed at a high level 47, 48. *Xenopus* frogs have a single classical class I gene along with the TAP (apparently at least oligomorphic) and tapasin genes located together, with this class I region between the class II region and the class III region [49]. Atlantic salmon have a single classical class I gene close to the TAP2 gene, this region having a strong genetic association with resistance to at least one economically important virus 50, 51.

The organization originally discovered for chickens is likely to be the ancestral one. The genes for antigen processing, peptide loading, and antigen presentation are not closely related so they did not evolve by gene duplication and acquisition of new functions. Instead, unrelated genes coevolved to work together as a pathway, and such coevolution is favored by close linkage. In other words, the genes of the class I system, and by extension the class II system, T cell receptors, and natural killer (NK) cell receptors, are likely to have emerged in one region, a primordial MHC, that has been falling apart ever since 6, 9. In support of this notion, genes found in various locations around the genome of mammals are found in or near the MHC of non-mammalian vertebrates. For example, the genes for an NK cell receptor and putative ligand (BNK and Blec, like NKR-P1 and LLT1/clr) are found in the chicken MHC rather than in the region syntenic to the NK complex (NKC) as in mammals 16, 52.

**Figure 3:**
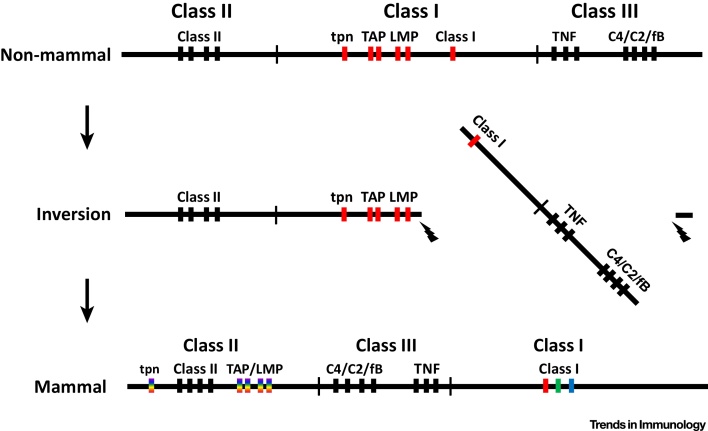
The Presence of a Multigene Family of Well-Expressed Classical Class I Molecules in Typical Placental Mammals Can Be Explained by a Genomic Inversion that Disrupted the Coevolutionary Relationships between the Closely Linked Genes of the Class I System Found in Many Other Vertebrates. Top panel. The genomic organization of an ancestral major histocompatibility complex (MHC) haplotype, based on data from the chicken and throughout the non-mammalian vertebrates, has class II genes in a class II region, class I genes and the genes encoding antigen-processing and peptide-loading components in a class I region, and the class III region genes on the outside. The close linkage within the class I region leads to a single dominantly expressed class I gene (red) whose peptide motif reflects the specificities of the polymorphic antigen-processing and peptide-loading genes (all red) with which it coevolves. Middle panel. A genomic inversion can lead to the class III region moving between the single dominantly expressed class I gene and the rest of the MHC, marooning the particular alleles of the antigen-processing and peptide-loading genes near the class II genes and far from the class I allele that they serve. Bottom panel. The antigen-processing and peptide-loading genes are selected to support any class I allele that might appear due to recombination (rainbow color), which would then allow duplication within an MHC haplotype to give a multigene family encoding class I molecules with different peptide motifs (red, green, blue), as is found in typical mammals. Regions separated by thin vertical lines; genes indicated by thicker vertical lines; tpn, tapasin; TAP, transporter associated with antigen presentation; LMP, inducible proteasome component, originally known as low molecular weight protein; C2, complement component 2; C4, complement component 4; fB, factor B. Modified from [9].

Thus, it is the mammalian MHC that is novel, and the MHC of at least one marsupial is organized like that of chickens [53], so the change occurred in the lineage leading to placental mammals 6, 9, 27. A potential mechanistic explanation for this change would be an inversion (Figure 3) that brought the class III region into the center of the MHC and swung the class I genes to the outside, with the breakpoint such that the antigen-processing and peptide-loading genes were left behind and eventually became part of the class II region. As discussed above, sufficient levels of recombination meant that advantageous combinations of genes could not stay together, and the TAP, tapasin, and inducible proteasome genes became average best fits for whatever class I allele appeared by recombination. Once many alleles could be serviced, a multigene family became possible.

## Low-Expression Promiscuous and High-Expression Fastidious Chicken Class I Alleles

Some associations with the chicken MHC could be easily explained by the BF2 class I allele from a resistant (but not a susceptible) chicken finding a protective peptide 37, 39, but the strong associations with Marek’s disease were more challenging to explain. Marek’s disease virus (MDV), an oncogenic herpes virus with a complex life cycle and significant evolution of virulence in historic times, has been an enormous economic problem [54]. It was unclear how the MHC might confer susceptibility, since any class I molecule would be expected to bind a protective peptide from at least one of the 100 MDV genes. However, MHC haplotypes like B19 were strongly associated with susceptibility while B2 and B21 were strongly associated with resistance [55].

It has become clear that the BF2 molecules from susceptible haplotypes have peptide motifs much like those of typical mammalian class I molecules, with several pockets in the binding groove each one of which binds only one or a few similar amino acids 37, 56. Such class I molecules might be called fastidious, with stringent peptide motifs and narrow peptide repertoires. By contrast, the BF2 molecules from resistant haplotypes can bind an exceedingly large variety of peptides and might be called promiscuous, with relaxed peptide motifs and wide peptide repertoires 38, 57. For instance, the molecule BF2*21:01 remodels the binding site to accommodate three anchor residues, at peptide positions P_2_, P_c-2_, and P_c_, with nearly every amino acid found at P_2_ and P_c-2_ 38, 57. Other chicken class I molecules, like BF2*02:01 and BF2*14:01, use broad binding pockets capable of accommodating many amino acids with hydrophobic side chains, which are particularly common in most proteins [57].

The correlation of peptide repertoire with resistance to Marek’s disease was unexpected. One hypothesis to explain this correlation [57] is that the few MDV peptides presented by fastidious molecules activate too few T cell clones to be effective (as seems to be the case based on one study [58]) while the promiscuous class I molecules provide a wide-ranging response involving many T cell clones. Alternatively, the truly protective peptides might be so few in number that the promiscuous BF2 molecules have a greater chance of presenting such peptides.

Intriguingly, the fastidious class I molecules are found on the cell surface at high levels whereas the promiscuous molecules are expressed on the cell surface at lower levels. This cell-surface expression level is not dependent on the level of transcription or translation or on the kinetics of translocation to the cell surface or degradation. Overall, the population of highly expressed fastidious molecules shows greater thermal stability than do the poorly expressed promiscuous molecules, although stable complexes with particular peptides are found for both kinds of molecule 35, 38, 57.

This inverse correlation of peptide repertoire with cell-surface expression was also unexpected. It seems most likely that the number of molecules arriving on the cell surface is determined by the interaction of the particular TAP, tapasin, and BF2 alleles in the peptide-loading complex. The underlying reason for this mechanism might simply be the biochemistry of peptide loading, but alternatively there could be evolutionary selection for the inverse correlation. One hypothesis is that promiscuous BF2 molecules present so many self-peptides that negative selection would deplete too many T cell clones in the thymus and that reducing cell-surface expression would reduce the extent of negative selection, with a balance of peptide repertoire and cell-surface expression resulting in an optimal T cell repertoire [57]. Several other hypotheses can be imagined, including a balance between the responses to pathogens for protection versus the recognition of self that could lead to autoimmunity, or the balance between antigen presentation for T cell recognition versus a role as a ligand for NK cells.

## An Inverse Correlation of Cell-Surface Expression and Peptide Repertoire for Human Classical Class I Alleles

To what extent do these observations about class I molecules extend beyond chickens? Obviously, the potential contributions of polymorphism in TAP and tapasin cannot extend to mammals that have monomorphic antigen-processing and peptide-loading genes. However, the linkages of peptide repertoire, cell-surface expression, translocation to the cell surface, stability, and resistance to a viral disease are found for human class I molecules. Striking differences were reported in the predicted peptide repertoire of four HLA-B alleles that correlated with the speed of progression from HIV infection to AIDS, with the fastidious HLA-B*57:01 and HLA-B*27:05 alleles associated with long-term non-progression compared with the promiscuous HLA-B*07:02 and HLA-B*35:01 associated with rapid progression [59]. Subsequently, the cell-surface expression levels of these four HLA-B alleles were shown to vary inversely with peptide repertoire, mirroring the findings in chickens [57]. Measurements of direct peptide binding for 27 HLA-A and HLA-B alleles showed a wide range of peptide repertoires [60]. An early immunoprecipitation study reported that one HLA-A and six HLA-B alleles were mostly in a peptide-bound conformation while seven HLA-A and three HLA-B alleles were mostly bound to TAP molecules, suggesting a range of peptide-loading efficiencies [61]. Assays with transfected cDNA clones for 27 HLA-B alleles show that some alleles have strong tapasin dependence on cell-surface expression (tapasin-independent alleles generally being correlated with faster HIV progression) [62], implicating dependence on translocation to the cell surface as in chickens [35]. Although the data are not strictly comparable between all of these reports, wider peptide repertoire, lower cell-surface expression level, longer TAP binding, tapasin independence of translocation, and faster HIV progression for HLA-A and HLA-B alleles seem to be broadly (but not perfectly) correlated [63], with many (but not all) HLA-A molecules being more promiscuous and many (but not all) HLA-B molecules being more fastidious. Overall, the similarities between chickens and humans suggest that these are fundamental properties of classical class I molecules.

However, there are clearly differences between the human and chicken class I systems. The range of peptide binding for human class I alleles appears to be less than that of chickens. For instance, HLA-A*02 molecules are the most promiscuous human class I molecules, accommodating hydrophobic amino acids that are common in proteins but only two or three different amino acids in each pocket as opposed to the six different amino acids accommodated by the highly promiscuous chicken BF2*02:01 57, 64, 65. Similarly, the fastidious HLA-B*57:01 specifies amino acids for only two pockets, one of which requires the rare amino acid tryptophan whereas the other allows the common amino acids alanine, serine and threonine. By contrast, the highly fastidious chicken class I molecule BF2*04:01 requires the binding of relatively rare acidic amino acids in each of three pockets 37, 56, 66. Perhaps the presence of a multigene family of human class I molecules means that the selective pressure for extremely promiscuous and fastidious molecules is lower than in chickens.

A second difference might be that cell-surface expression has been correlated with tapasin dependence in humans but thus far only with TAP specificity in chickens; the effect of chicken tapasin has not been examined 35, 62. In any case, human tapasin and TAP genes are functionally monomorphic, so any effect in the peptide-loading complex would depend on the polymorphic positions in the class I allele 42, 43, 44. By contrast, chicken tapasin and TAP genes are all polymorphic and appear to coevolve with the dominantly expressed class I BF2 gene 26, 27, so the interactions could be more complex.

HLA-C presents a special challenge, perhaps because the relative importance of various sequence features remains controversial. HLA-C molecules are found expressed on the surface of most cells at a much lower level than HLA-A and HLA-B (perhaps commensurate with a greater role for HLA-C as a ligand for NK cells rather than as an antigen presentation molecule for T cells) [67]. In addition, HLA-C alleles vary in their cell-surface expression (with higher expression correlated with slower HIV progression, perhaps due to T cell recognition) 14, 68, 69. Various features of HLA-C have been reported to contribute to these two kinds of differences, including: promoter sequence and transcription; miRNA sites in the 3′ UTR sequences and RNA stability; β_2_m association, peptide motif, and peptide repertoire; and TAP residency and translocation to the surface 67, 70, 71, 72, 73, 74, 75, 76. In early studies 61, 70, certain HLA-C alleles were found to be present inside the cell at the same level as HLA-A and HLA-B molecules but remained bound to TAP and were not translocated to the cell surface, similar to promiscuous chicken class I alleles. The available data from predicted or actual peptide motifs is often interpreted to show a limited number of peptides that can bind HLA-C molecules compared with typical HLA-A and HLA-B molecules 67, 74, 75, 76. A more recent report [76] compares two HLA-C alleles, confirming that several features of the HLA-C gene contribute in a complex way to cell-surface expression. The peptide-binding domains of one HLA-C allele that binds a greater diversity of peptides are better expressed at the cell surface (at least when fused to another class I molecule), the opposite of that found for chicken class I molecules. How all of these observations fit together is currently unknown.

Finally, perhaps the most striking difference is that poorly expressed promiscuous alleles confer protection against Marek’s disease in chickens while well-expressed fastidious alleles are responsible for slow progression to AIDS in humans. Any pretense to an overarching model must explain this difference.

## Promiscuous Generalists and Fastidious Specialists

**Figure 4:**
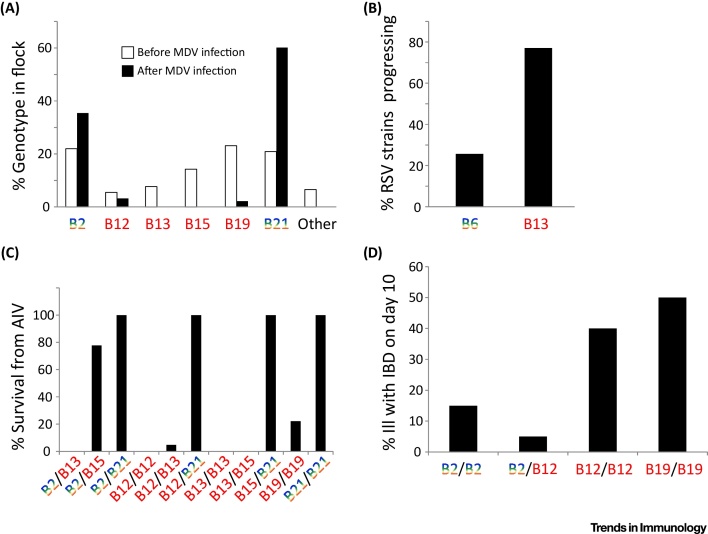
Chicken Major Histocompatibility Complex (MHC) Haplotypes Encoding Promiscuous Class I Molecules (Rainbow) Can Confer Protection against a Variety of Viral Infections Under Experimental and Field Conditions, Whereas MHC Haplotypes Encoding Fastidious Class I Molecules (Red) Generally Confer Susceptibility. Percentage of MHC genotypes in a flock before and after experimental infection with Marek’s disease virus (MDV), with the B2 and B21 haplotypes conferring protection (A). Percentage of Rous sarcoma virus (RSV) strains that progress to give lethal tumors after experimental infection, with the B6 haplotype conferring survival (B). Percentage survival after natural infection with avian influenza virus (AIV) under field conditions in rural Thailand, with the presence of a single promiscuous haplotype conferring protection, except in one combination (B2/B13) for reasons that are not understood (C). Percentage of chickens ill from infectious bronchitis virus (IBV) on day 10 after experimental infection, with the B2 haplotype conferring protection (D). Data from 77, 78, 79, 80.

What could be the evolutionary basis for having well-expressed fastidious and poorly expressed promiscuous class I alleles? Looking through the literature, it appears that the promiscuous BF2 alleles protect chickens against a range of common infectious diseases in addition to Marek’s disease 77, 78, 79, 80. Among several examples (Figure 4), typing chickens in rural Thailand after an outbreak of avian influenza found that all B21 homozygotes survived, that all chickens homozygous for the B12, B13, and B15 haplotypes with fastidious BF2 molecules died, and that, in all but one combination, heterozygotes with one promiscuous class I allele survived [79]. It appears that promiscuous BF2 molecules, combining the specificities of several fastidious molecules in one molecule, generally confer protection against most pathogens (Figure 2), much like a mammalian MHC haplotype with multiple mammalian class I molecules. By contrast, the fastidious human class I alleles HLA-B*57:01 and HLA-B*27:05 confer protection against the dangerous zoonotic pathogen HIV, which they achieve by binding a particular protective peptide that the virus cannot change without a drastic loss of fitness 13, 59, 81, 82. Another dangerous and possibly new pathogen, hepatitis C virus (HCV), is also controlled by HLA-B*27:05 83, 84.

**Figure 5:**
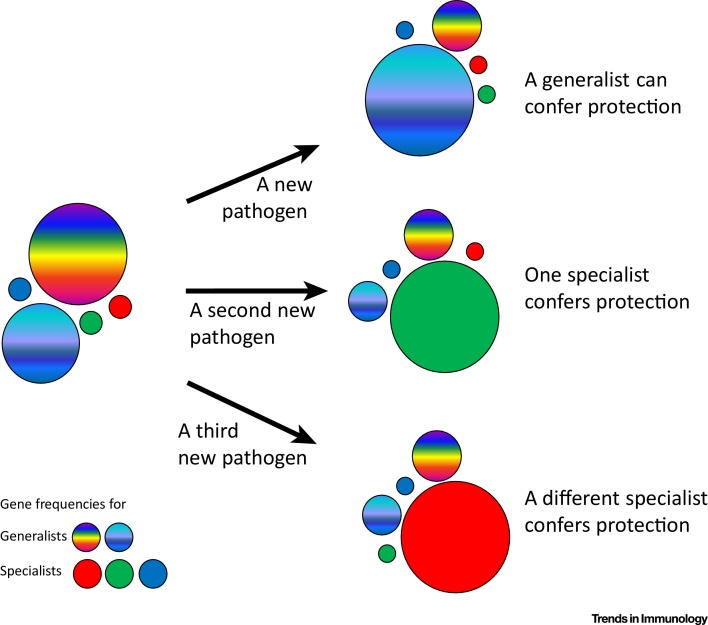
A Model Illustrates the Shift in Gene Frequencies from A Few Predominant Generalist Major Histocompatibility Complex (MHC) Alleles on Selection by New and/or Particularly Virulent Pathogens. The diameter of each circle indicates the frequency of a particular MHC allele in a population before and after selection by a pathogen. The rainbow colors indicate promiscuous molecules that act as generalists, conferring protection against most pathogens including those regularly found in the environment. The single colors indicate fastidious molecules encoded by genes that arise by mutation and are present at low frequency but with the possibility of presenting a protective peptide from a particular pathogen. Scenarios for three different pathogens are shown: one of the generalist molecules confers protection against the first pathogen (top); one of the specialist molecules confers protection against the second pathogen (middle); and another of the specialist molecules confers protection against the third pathogen (bottom). Modified extensively from [63].

Putting these two ideas together, one evolutionary hypothesis would be that low-expression promiscuous class I alleles function as generalists while the high-expression fastidious alleles act as specialists 57, 63. Most of the time, the generalists deal well with common pathogens, but they may not always be able to cope with the appearance of a new and virulent pathogen. In this case, a particular fastidious molecule may present a special peptide from the new pathogen, conferring protection and leading to an increase in gene frequency for that allele (Figure 5).

How does this new view stack up against the current concepts and available data? The many class I alleles found in most human populations have long been interpreted to mean that high levels of polymorphism are important for survival. However, a population with a few generalist alleles may provide enough protection under normal circumstances. If this is true, the current view that MHC typing can identify the risk of extinction for endangered species [85] may need to be reconsidered to also take into account the peptide repertoire of MHC molecules present in the remaining population. It is also easy to imagine that the original class I alleles were promiscuous, which seems superficially similar to the class II molecules from which they may have arisen 6, 9, 86. The major change in structure–function relationships caused by rearrangement of the MHC in the lineage leading to placental mammals discussed above 6, 9, 27 might have been facilitated by a promiscuous class I allele closely linked to promiscuous TAP genes.

Fastidious alleles might arise by a few mutations from promiscuous alleles and remain at a low gene frequency unless selected by a pathogen challenge and might diminish in frequency once that challenge is relaxed. However, it is also possible that a selective sweep ensures the near fixation of fastidious alleles. Chimpanzees, which are thought to have been strongly selected during a retroviral catastrophe, have two kinds of class I molecules 87, 88, 89: those with fastidious peptide motifs similar to HLA-B*57:01 and HLA-B*27:05 and those with promiscuous motifs similar to BF2*02:01. Finally, it is important to note that the classification of generalist and specialist alleles may be more useful for the explanation of a population response to a given pathogen than for individual predictions: a promiscuous molecule may be able to protect against a new and virulent pathogen (as does HLA-B*35:01 for HIV clade C but not B [13]) while most fastidious molecules will be unlikely to recognize a protective peptide for any one particular pathogen. However, a potential advantage might arise from bundling together alleles that are promiscuous or fastidious, allowing greater statistical power in genetic association studies.

## Concluding Remarks

The observations and hypotheses described in this review require much additional work for support and testing (see Outstanding Questions). First, careful and quantitative measurement of peptide repertoire breadth, cell-surface expression levels, and translocation to the surface for class I alleles in homozygotes is required. If the broad correlations discussed here are confirmed, a comprehensive reassessment of the extent to which (low-expression) promiscuous and (high-expression) fastidious class I alleles confer protection against various kinds of pathogens (as well as correlating with other biological and medical phenomena) would be valuable.

A much deeper understanding of the mechanisms underlying the phenomenon is clearly required. If a fundamental property leads to these correlations for classical class I molecules, it is natural to ask whether the same phenomenon may be true for classical class II molecules. The concept of generalist and specialist has already been used for class II genes in relation to the nematode burden of striped mice in Africa [90]. Perhaps the same idea of promiscuous and fastidious recognition might be true for other (particularly innate) immune receptors.

Finally, much can be learned from evolutionary biology approaches, including observation and simulation, typically with wild outbred populations. For detailed disease associations including autoimmunity and for mechanistic studies, humans and mice are obviously much better suited for rapid progress than chickens. However, at the least, the chicken MHC again has provided a simple model to discover phenomena that have been difficult to discern in both the more complicated MHC of typical mammals and the less well-characterized MHC of wild species.

Outstanding Questions To what extent is the inverse correlation between cell-surface expression and peptide repertoire found for all classical class I molecules in chickens and humans? Is it a true hierarchy or just two groups? If this is not the case for human HLA-C, why?

What is the mechanism for the inverse correlation between cell-surface expression and peptide repertoire found for classical class I molecules? Is it due to intrinsic differences in the folding of the class I molecule, the efficiency of interactions with the peptide-loading complex, quality control steps (like TAPBPR/UGT), or other important steps of translocation? In addition to these biochemical mechanisms, what are the selective pressures for the inverse correlation between cell-surface expression and peptide repertoire found for classical class I molecules (e.g., optimization of T cell repertoire, avoidance of autoimmunity)?

To what extent does low expression level/peptide promiscuity really correlate with resistance to common pathogens? How is this correlation influenced by the type of pathogen involved (e.g., viruses with small or large genomes, for which the numbers of potential protective peptides differ) and what is the underlying mechanism for protection (number of T cells activated, higher probability of binding a given efficacious peptide)?

Similarly, what are the mechanisms underlying protection against a given zoonosis by high-expression level/fastidious peptide-binding MHCs? Is the binding of special protective peptides that are critical for viral fitness always involved?

To what extent is this new view true for a wide variety of species? These studies should be easier in mice and primates where a significant literature is available but are largely relevant across the animal kingdom (e.g., farm and sport animals, farmed fish).

To what extent are these features found for classical class II molecules?
